# Analysis of the clinical diagnostic efficacy of the novel serum markers lipase F, gastrokine 2 and the collagen X-type α1 chain in gastric cancer

**DOI:** 10.5937/jomb0-59476

**Published:** 2026-02-27

**Authors:** Fashe Wang, Bai Li, Shangyin Li, Yan Tang, Tan Tao

**Affiliations:** 1 Ningbo Mingzhou Hospital, Department of General Surgery Zhejiang Province, Ningbo, China; 2 Fujian Medical University First Affiliated Hospital, Department of Gastroenterology, Fuzhou, China; 3 Third People's Hospital of Hubei Province, Department of Gastroenterology, Wuhan, Hubei, China

**Keywords:** serum markers, lipase F, gastrokine 2, collagen X-type α1 chain, gastric cancer, serumski markeri, lipaza F, gastrokin 2, kolagen tipa X α1 lanac, karcinom želuca

## Abstract

**Background:**

To explore the application value of three novel serum markers, collagen X-type <span style="color: rgb(32, 33, 34); font-family: sans-serif; font-size: 16px; background-color: rgb(248, 249, 250);">α</span>1 chain (COL10A1), gastrokine 2 (GKN2), and lipase F (LIPF), in the diagnosis of gastric cancer.

**Methods:**

Differential markers of gastric cancer were mined from public databases (TCGA, GTEx). From November 2022 to October 2024, 108 healthy people and patients with stomach cancer who underwent gastroscopy and pathological examinations at our hospital were chosen as research subjects. Among these patients, 60 had stomach cancer (28 in the early gastric cancer group and 32 in the advanced gastric cancer group), whereas 48 patients were in the healthy group. Receiver operating characteristic (ROC) curves were created to examine the relationship between the detection of gastric cancer and the serum levels of markers. Additionally, the application value of both the detection of new markers and the detection of conventional markers (PGI, PGII) in the diagnosis of gastric cancer was examined.

**Results:**

Three differential markers of gastric cancer (COL10A1, GKN2 and LIPF) were extracted from a public database. By detecting the levels of three markers in clinical blood samples, the AUC of the combined detection of the three markers (COL10A1+ GKN2 + LIPF 3MP) in the differentiating group (3MP vs PGR: 0.842 vs 0.841) and the early gastric cancer group (0.877 vs 0.843) was greater than that of PGR (PGI/PGII). The combined conventional markers (PGI, PGII) and the combined detection of five markers (3MP+PGI + PGII, 5MP) had AUCs of 0.963 and 0.953, respectively.

**Conclusions:**

COL10A1, GKN2 and LIPF are very promising serum markers for the diagnosis of gastric cancer. The joint detection of the serum levels of these three novel markers and PGI and PGII has essential value for the early detection of gastric cancer.

## Abstract (sr)

**Uvod:**

Cilj e ispita dijagnostička vrednost tri nova serumska markera - kolagen tipa X <span style="color: rgb(32, 33, 34); font-family: sans-serif; font-size: 16px; background-color: rgb(248, 249, 250);">α</span>1 lanac (COL10A1), gastrokina 2 (GKN2) i lipaze F (LIPF) - u dijagnostici karcinoma želuca.

**Metode:**

Diferencijalni markeri za karcinom želuca su identifikovani iz javno dostupnih baza podataka (TCGA, GTEx) Za ispitanike je izabrano 108 zdravih osoba i pacijenata sa karcinomom želuca koji su u našoj ustanovi podvrgnuti gastroskopiji i patohistološkim analizama od novembra 2022. do oktobra 2024. godine. Od toga je 60 pacijenata imalo karcinom želuca (28 u grupi sa ranim karcinomom i 32 u grupi sa uznapredovalim karcinomom), dok je 48 osoba činilo zdravu kontrolnu grupu. Krive operativne karakteristike (ROC krive) su korišćene za analizu povezanosti između nivoa seruma markera i prisustva karcinoma želuca. Takođe je ispitivana dijagnostička vrednost novih markera u poređenju sa konvencionalnim markerima (PGI, PGII).

**Rezultati:**

Tri diferencijalna markera karcinoma želuca (COL10A1, GKN2 i LIPF) izdvojena su iz javnih baza podataka. Detekcijom njihovih nivoa u kliničkim uzorcima krvi, AUC kombinovane detekcije ova tri markera (COL10A1+ GKN2 + LIPF 3MP) bio je veći u diferencijalnoj grupi (3MP naspram PGR: 0,842 naspram 0,841) i u grupi sa ranim karcinomom želuca (0,877 naspram 0,843) u poređenju sa PGR (PGI/PGII). Kombinacija konvencionalnih markera (PGI, PGII) i nova kombinacija svih pet markera (3MP+PGI + PGII, 5MP) imale su AUC vrednosti od 0,963 i 0,953, redom.

**Zaključak:**

COL10A1, GKN2 i LIPF predstavljaju serumske markere za dijagnozu karcinoma želuca koji veoma obećavaju. Zajednička detekcija nivoa ova tri nova markera zajedno sa PGI i PGII ima značajnu vrednost za rano otkriće karcinoma želuca.

## Introduction

Globally, gastric cancer ranks as the third most common cause of death for cancer patients, with over 350,000 cases and more than 260,000 deaths each year [1]. Surgery and chemotherapy are the main treatment methods, but the 5-year survival rate of patients in the advanced stage is only 6% [2]. Early diagnosis of gastric cancer can increase the cure rate. Still, its early symptoms are not obvious and are similar to those of benign diseases such as gastritis, resulting in a low diagnosis rate [3]. Despite being the most effective screening technique at present, gastroscopy has a low compliance rate. Therefore, the development of noninvasive detection methods is crucial for the management of gastric cancer.

One popular noninvasive cancer screening technique is serum protein marker screening. Its non-invasiveness, affordability, and ease of use make it a popular choice for clinical testing and physical examinations. These markers are helpful for early warning, diagnosis, severity assessment, treatment guidance, recurrence and metastasis monitoring, and prognosis assessment of gastric cancer [4]. The »Expert Consensus on Screening and Testing Techniques for Early Gastric Cancer« [5] suggests conducting pepsinogen I (PGI) and pepsinogen II (pepsinogen II) tests for high-risk populations of patients with gastric cancer II. PGII, gastrin-17 (G-17), PGR (PGI/PGII), and *Helicobacter pylori *(HP) were detected. However, relevant studies [6] reported that the sensitivities of PGI, PGII, and PGR were all lower than 70%, and the specificities were all lower than 50%. In addition, several traditional markers, such as carcinoma embryonic antigen (CEA), carbohydrate antigen 19-9 (CA19-9), and G-17, are abnormally elevated in patients with acute hepatitis, cholelithiasis, and cholangitis [7][8][9]. Serum markers are prone to false positives and cause anxiety in patients. Therefore, the search for new biomarkers is crucial for the early diagnosis of gastric cancer.

This method is used for the early diagnosis of gastric cancer. This study screened these three candidate markers from the TCGA and GTEx databases and conducted clinical tests among 108 people who underwent gastroscopy and pathological examinations. This study compared the diagnostic performance of three new markers and their combinations (3MP) and the five-marker combination combined with the traditional markers PGI and PGII (5MP) in patients with different stages of gastric cancer and compared them with those of the conventional markers PGI, PGII and PGR (PGI/PGII).

This study aims to provide a scientific basis for the early diagnosis of gastric cancer and promote the clinical management of early gastric cancer.

## Materials and methods

### Data collection

Sixty patients with stomach cancer (gastric cancer group) and 48 healthy people who had physical exams (healthy group) who were admitted to our hospital between November 2022 and October 2024 were chosen as the clinical test subjects. Among these patients, 32 had advanced stomach cancer (advanced gastric cancer group), and 28 had early gastric cancer (early gastric cancer group).

One millilitre of blood was drawn from 108 participants. After separation, the serum was stored at -80°C until use. Additionally, all recruited participants' clinical data, such as age, sex, HP test results, tumour size, TNM stage, tumour stage, pathological type, PGI serum level, PGII serum level, and prior illness history, were documented.

### Inclusion criteria and exclusion criteria

The inclusion criterion for the gastric cancer group was patients with gastric cancer diagnosed by gastroscopy and clinicopathological examination. Patients with other malignant tumours or autoimmune diseases, and patients whose clinical information was incomplete. The healthy group included subjects who were diagnosed by gastroscopy as having no atrophic lesions or polyps in the stomach, had negative HP test results, and had no major diseases or tumours during physical examination.

### Database mining

For bioinformatics research to evaluate possible markers of early gastric cancer, data from patients with gastric cancer and healthy controls were sourced from public sources. UCSC Xena (HTTPS://xena-browser.net/datapages/) was used to obtain healthy people from GTEx RNAseq, and the clinical data of patients with gastric cancer were obtained from the TCGA data from CPTAC (HTTPS://cptac-data-portal.georgetown.edu/). The gene and protein expression levels of gastric cancer and normal tissues were compared, and the differentially expressed genes and proteins (genes: Log2|FC|≥2, FDR>0.01) were screened. Proteins: Log2|FC|≥1, FDR>0.05. Go and KEGG analyses were conducted on the top 10 DEGs. On the basis of the comprehensive analysis of the results, protein expression, secretion properties and procurement of reagent kits, candidate markers suitable for performance testing were identified.

### Determination of the serological levels of markers

An enzyme-linked immunosorbent assay was used to measure the serum marker levels of COL10A1, GKN2, and LIPF. COL10A1 was tested via a human collagen X enzyme-linked immunosorbent assay kit (Novus Biologicals, NBP2-75826), GKN2 was tested via a human stomach factor 2 enzyme-linked immunosorbent assay kit (Novus Biologicals, NBP3-08145), and LIPF was assessed via a Circumferric Lipase Enzyme-Linked Immunosorbent Assay Kit (Novus Biologicals, NBP3-00409). A chemiluminescence platform was used to evaluate the serum levels of PGI and PGII. PGI uses the pepsinogen I assay kit (Mindray, 220801), whereas PGII uses the pepsinogen II assay kit (Mindray, 220401).

### Statistical methods

SPSS 25.0 was used for the statistical analysis. Independent sample t tests were used to compare two groups, and the means ± standard deviations are expressed for continuous data with a normal distribution. The Mann Whitney U test was used to compare two groups, and medians [M(Q1, Q3)] were used to indicate nonnormally distributed data. The 2 test was used to compare two groups, and categorical data are represented as n (%). ANOVA, or one-way analysis of variance, was used for comparisons between groups. The relationships between markers and clinical traits were examined via Spearman's rank correlation analysis. There was a positive correlation when r>0 and a negative correlation when r<0. The association was more significant when |r| was greater. The indicators' combined diagnostic performance was examined via a logistic regression model. GraphPad Prism 8 and MedCalc 16.8.4 were used to create receiver operating characteristic (ROC) curves. The diagnostic value was assessed via the area under the curve (AUC), sensitivity, and specificity. Statistical significance was defined as a P value <0.05.

## Results

### Database mining of candidate markers

**Figure 1:**
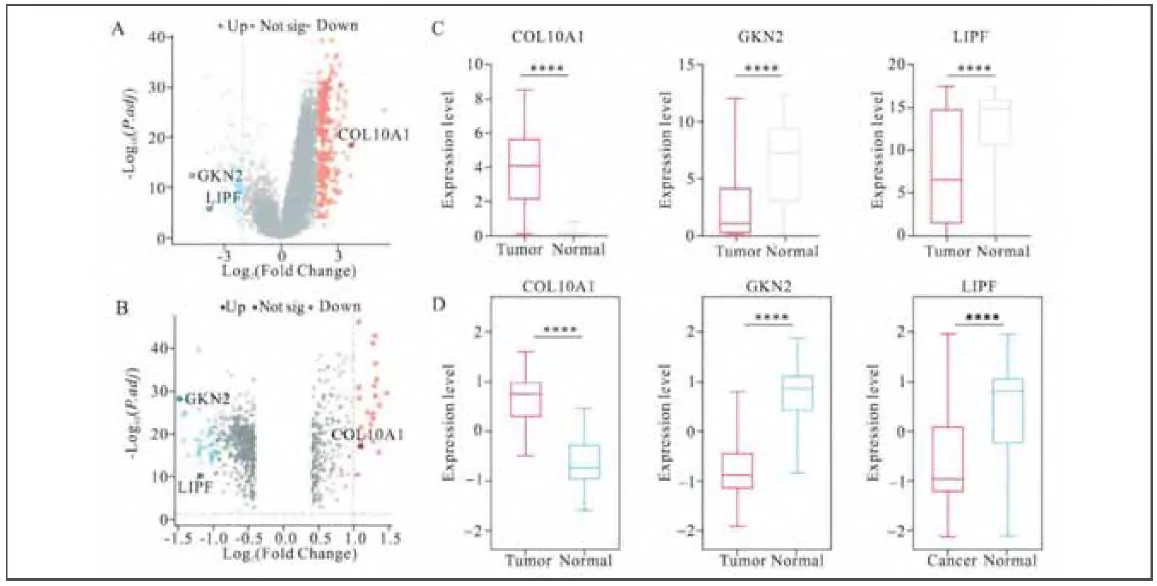
The TCGA, GTEx, and CPTAC databases revealed that the gastric cancer group and the healthy group presented distinct gene and protein expression patterns.<br>A: Volcano maps of genes that are expressed differently in healthy tissues and stomach cancer, derived from the TCGA and GTEx databases; B: Volcano maps of the proteins that distinguish stomach cancer from healthy tissues via the CPTAC database; C: Gene expression levels of three potential markers derived from the TCGA and GTEx databases in stomach cancer and healthy tissues; D: Protein expression levels of three potential markers derived from the CPTAC database in stomach cancer and healthy tissues.

Differential expression analysis, diagnostic performance analysis, and survival curve analysis were performed using data from 734 samples (359 healthy samples and 375 gastric cancer samples) in the TCGA and GTEx databases, combined with the location information of proteins and the procurement situation of scientific research kits. Finally, we identified three markers that were significantly differentially expressed in gastric cancer and suitable for in vitro performance testing, namely, COL10A1, GKN2 and LIPF, as shown in Figure 1.

### Analysis of clinical test cohort characteristics

To explore the diagnostic performance of COL10A1, GKN2 and LIPF in clinical samples, we collected serum samples from 108 patients who visited our hospital from November 2022 to October 2024. The clinical baseline information is shown in Table 1. Among the 108 patients, 48 were in the healthy control group. They underwent physical examinations in the hospital (healthy group), and 60 patients were diagnosed with gastric cancer by gastroscopy and pathological diagnosis (gastric cancer group). Forty-two patients (39%) and 66 patients (61%) were female out of the 108 patients. Eighty-nine (82%) of the patients were older than forty years. Ninety-eight per cent of the patients in the stomach cancer group were older than 40 years. Forty-two per cent of the HP samples were positive. Among them, 28 patients (47%) had early gastric cancer, and 32 patients (53%) had advanced gastric cancer. There were 12 patients (20%) with tumours larger than 3.84 cm. There were 34 patients (57%) with the pathological type of adenocarcinoma. There were 21 cases (35%) of nerve invasion and 19 cases (32%) of vascular invasion. Among the previous medical records, 29 patients (48%) had hypertension, 9 patients (15%) had diabetes, and 22 patients (37%) had other diseases (gallbladder stones, cerebral infarction, pulmonary nodules).

**Table 1:** Clinical baseline information of 108 individuals, n (%)

Variables	Healthy (n=48)	Gastric cancer
Gender		
Male	22 (46)	44 (73)
Female	26 (54)	16 (27)
Age (years)		
≥40	30 (63)	59 (98)
<40	18 (38)	1 (2)
HP		
Positive	0 (0)	25 (42)
Negative	48 (100)	35 (58)
PGI (ng/mL)		
≥76.61	11 (23)	24 (40)
<76.61	37 (77)	36 (60)
PGII (ng/mL)		
≥15.41	4(8)	32 (53)
<15.41	44 (92)	28 (47)
PGR		
≥5.98	35 (73)	19 (32)
<5.98	13 (27)	41 (68)
TNM		
I	-	23 (38)
II	-	5 (8)
III	-	23 (38)
IV	-	9 (15)
Stage		
Early	-	28 (47)
Advanced	-	32 (53)
Tumour size(cm)		
≥3.84	-	12 (20)
<3.84	-	36 (60)
Unknown	-	12 (20)
Pathological type		
Adenocarcinoma	-	34 (57)
Other	-	26 (43)
Invasive		
Nerve	-	21 (35)
Vascular	-	19 (32)
No	-	20 (33)
Diseases history		
Hypertension	-	29 (48)
Diabetes	-	9 (15)
Other	-	22 (37)

### Comparison of the serum levels of various markers

To explore the feasibility of COL10A1, GKN2 and LIPF as diagnostic markers for gastric cancer, this study detected and compared the serum levels of these three new markers with those of two conventional gastric cancer markers, PGI and PGII, in different groups (Table 2). There was a significant difference (P<0.001) in the mean blood levels of COL10A1, GKN2, LIPF, and PGII between the groups with gastric cancer (GC), early gastric cancer (EGC), advanced gastric cancer (AGC), and the healthy group. The gastric cancer, early gastric cancer, and advanced gastric cancer groups had significantly higher serum levels of COL10A1, GKN2, LIPF, and PGII than did the healthy group (P<0.001). However, the mean blood levels of PGI did not differ significantly between the groups with advanced gastric cancer, early gastric cancer, and gastric cancer and the healthy group (P=0.214). These results indicate that COL10A1, GKN2, and LIPF may be employed as markers for the identification of gastric cancer since they are highly expressed in the serum of patients with this disease.

**Table 2:** Comparison of serum levels of biomarkers in healthy group, GC group, EGC group and AGC group (x̄±s).

Group	COL10A1	GKN2	LIPF	PGI	PGII
Healthy (n=48)	0.41±0.47	3.65±0.77	5.95±0.71	65.86±46.58	8.64±5.20
GC (n = 60)	1.07±0.55^a^	4.44±0.47^a^	6.47±0.49^a^	84.71±51.24	20.51±15.64^a^
EGC (n = 28)	1.13±0.56^a^	4.45±0.40^a^	6.51±0.38^a^	82.18±43.48	17.55±8.16^a^
AGC (n=32)	1.02±0.54^a^	4.44±0.52^a^	6.44±0.57^a^	86.84±57.59	22.98±19.68^a^
F	18.063	21.965	9.570	1.510	8.725
P	0.000	0.000	0.000	0.214	0.000

### Correlation analysis of biomarker levels and the incidence of gastric cancer

**Figure 2:**
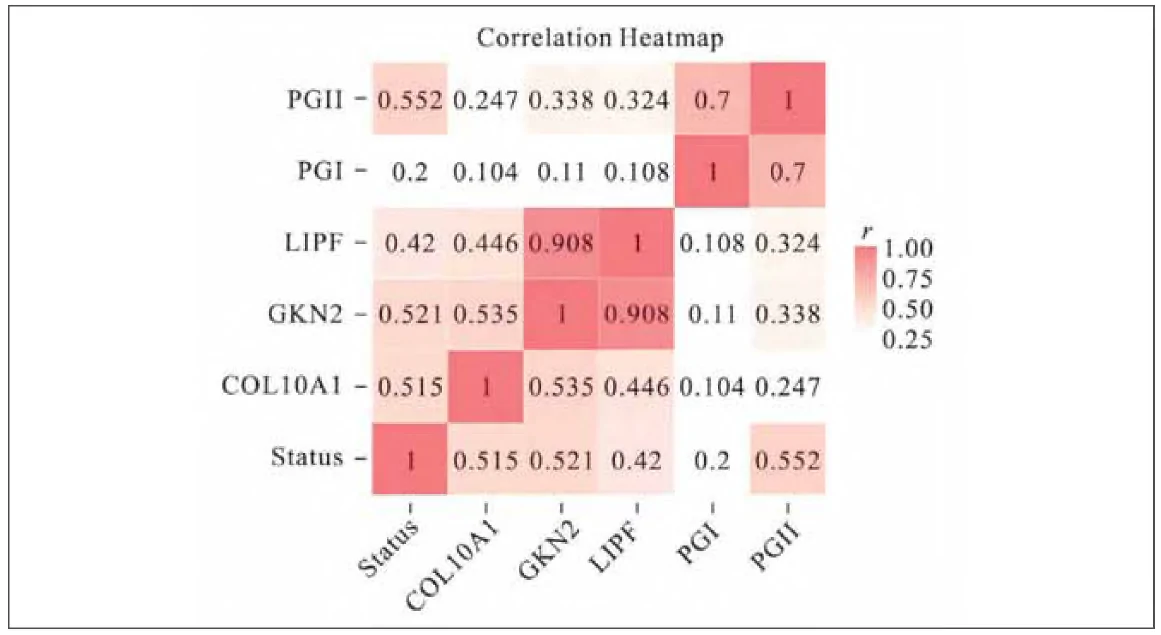
Health illustrating the correlation between illness status and biomarkers.

Spearman rank correlation analysis was used to explore the correlations between the disease status of the subjects (healthy, early gastric cancer and advanced gastric cancer) and the concentration levels of the five biomarkers. As shown in Figure 2, the serum concentrations of the five markers were positively correlated with the disease status of the subjects. The serum concentrations of COL10A1, GKN2 and PGII were significantly correlated with the disease status of the subjects (r>0.5), whereas LIPF and PGI were only weakly correlated (r<0.5). There was also a significant positive correlation among COL10A1, GKN2 and LIPF. Among them, COL10A1 was significantly and strongly correlated with GKN2, and LIPF was significantly and strongly correlated with GKN2 (r>0.5).

### The application of single biomarker detection in the diagnosis of gastric cancer

**Figure 3:**
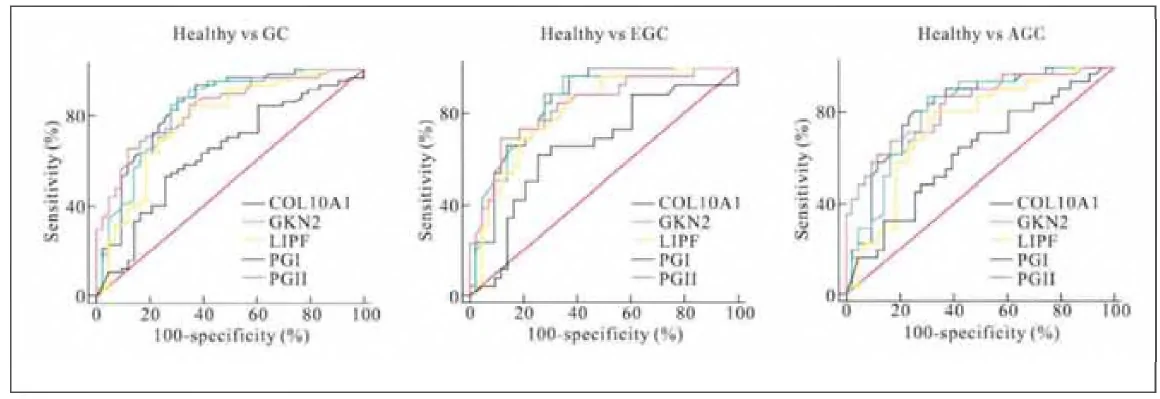
ROC curves for gastric cancer biomarker diagnosis.

The performance of COL10A1, GKN2 and LIPF in the diagnosis of gastric cancer was analysed via receiver operating characteristic (ROC) curves and compared with the performance of the conventional gastric cancer markers PGI and PGII, as shown in Figure 3. When the healthy group was differentiated from the gastric cancer group, the AUCs of COL10A1, GKN2 and LIPF all reached above 0.75. Among them, the AUC of COL10A1 was the highest (0.834, 95% CI: 0.749-0.918), which was greater than that of PGII. GKN2 (0.822, 95% CI: 0.735-0.908) and LIPF (0.775, 95% CI: 0.679-0.871) were slightly lower than PGII. For early diagnosis, the AUCs of COL10A1, GKN2 and LIpF can all reach above 0.8, and the detection sensitivities are all higher than those of PGI and PGII (Table 3).

**Table 3:** Comparison of the performance of 5 biomarkers in the diagnosis of gastric cancer.

Group	Biomarker	AUC	P	95% CI		Sensitivity<br>(%)	Specificity<br>(%)
				Low	High		
Healthy vs GC	COL10A1	0.834	0.000	0.749	0.918	85.00	68.75
	GKN2	0.822	0.000	0.735	0.908	85.00	75.00
	LIPF	0.775	0.000	0.679	0.871	85.00	64.58
	PGI	0.636	0.016	0.525	0.746	52.60	74.40
	PGII	0.830	0.000	0.752	0.909	64.91	88.37
Healthy vs EGC	COL10A1	0.847	0.000	0.758	0.937	89.29	68.75
	GKN2	0.841	0.000	0.746	0.936	89.29	75.00
	LIPF	0.812	0.000	0.71	0.915	89.29	64.58
	PGI	0.652	0.030	0.515	0.788	61.54	74.42
	PGII	0.831	0.000	0.731	0.932	69.23	88.37
Healthy vs AGC	COL10A1	0.822	0.000	0.725	0.918	84.37	66.67
	GKN2	0.806	0.000	0.706	0.905	87.50	70.83
	LIPF	0.743	0.000	0.630	0.857	81.25	68.75
	PGI	0.622	0.064	0.493	0.752	64.52	58.14
	PGII	0.829	0.000	0.735	0.924	67.74	83.72

### The application of combined detection of markers in the diagnosis of gastric cancer

**Figure 4:**
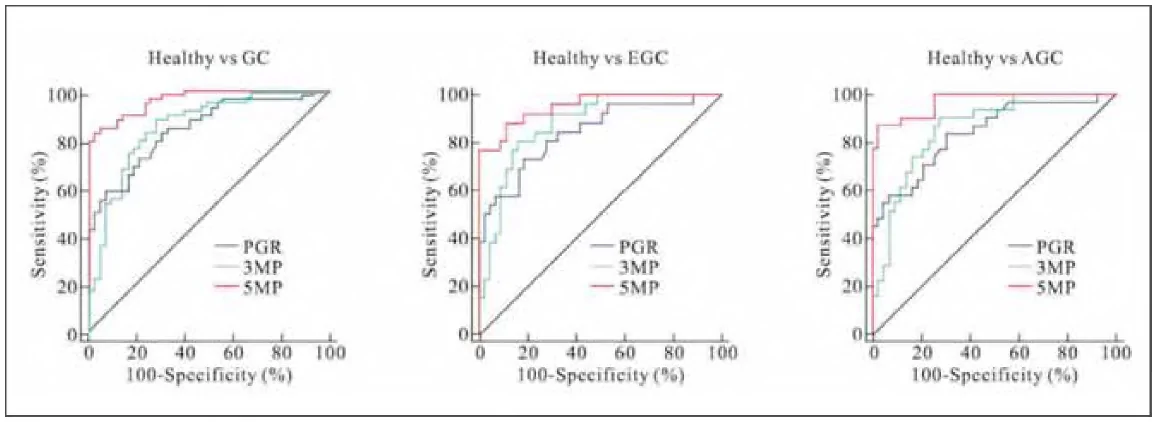
The PGR, 3Mp and 5MP ROC curves for gastric cancer diagnosis.

To further verify the application prospects of COL10A1, GKN2 and LIPF in the diagnosis of gastric cancer, in this study, a three-in-one test (COL10A1+GKN2 + LIPF, 3MP) and a five-in-one test (3MP+PGI + PGII, 5MP) diagnostic model were constructed through a logistic regression model. It was compared with the performance of PGR (PGI/PGII), which is routinely used in clinical practice. The ROC curve is shown in Figure 4. As shown in Table 4, the AUC of gastric cancer detection with 3MP was 0.842, the sensitivity was 85%, and the specificity was 79%. The AUC for the detection of early gastric cancer reached 0.877, with a sensitivity of 82.14% and a specificity of 85.42%. The AUC for advanced gastric cancer detection was 0.859, with a sensitivity of 90.62% and a specificity of 75.00%.

**Table 4:** Comparison of the performance of PGR,3MP and 5MP for gastric cancer diagnosis.

Group	Biomarker	AUC	P	95% CI		Sensitivity<br>(%)	Specificity<br>(%)
				Low	High		
Healthy vs GC	PGR	0.841	0.000	0.765	0.916	82.46	69.77
	3MP	0.842	0.000	0.760	0.924	85.00	79.00
	5MP	0.963	0.000	0.905	0.990	82.46	97.67
Healthy vs EGC	PGR	0.843	0.000	0.745	0.941	73.08	81.40
	3MP	0.877	0.000	0.796	0.957	82.14	85.42
	5MP	0.953	0.000	0.873	0.989	76.92	100.00
Healthy vs AGC	PGR	0.841	0.000	0.749	0.933	83.87	69.77
	3MP	0.859	0.000	0.775	0.943	90.62	75.00
	5MP	0.969	0.000	0.900	0.995	87.10	97.67

When the five markers were detected together, the diagnostic performance of 5MP was significantly improved. When detecting gastric cancer, the AUC of 5Mp was 0.963, which was 14.37% greater than that of 3MP [(AUC5MP-AUC3MP)/AUC3MP; the calculation method was the same as that in the following text]. When early gastric cancer was detected, the AUC was 0.953, increasing by 8.67%. When detecting advanced gastric cancer, the AUC was 0.969, rising by 12.81%. Among the three groups of detections, the detection sensitivity of 5MP was greater than 75%, and the specificity was greater than 97%. Combined detection can supplement the multidimensional detection performance of individual markers. Moreover, by jointly detecting three markers, PGI and PGII, the detection performance of 3MP and PGR can be further improved, significantly improving the detection efficiency for gastric cancer.

## Discussion

The 5-year survival rate of patients with stage I gastric cancer exceeds 90%, whereas that of patients with stage IV gastric cancer accompanied by distant metastasis is less than 6% [10][11]. Therefore, early diagnosis is crucial for curing gastric cancer. However, early-stage gastric cancer has no noticeable symptoms and is difficult to detect and attract attention [12]. At present, endoscopy combined with pathological examination is the gold standard for diagnosing gastric cancer, but it is not suitable for large-scale screening [13]. Therefore, it is necessary to develop effective noninvasive examination methods for the clinical management of gastric cancer. In this study, through database mining, three biomarkers suitable for the diagnosis of gastric cancer (COL10A1, GKN2 and LIPF) were screened out, and their performance in the early diagnosis of gastric cancer was verified in clinical samples and compared with that of PGI and PGII.

This study revealed that COL10A1, GKN2 and LIPF have excellent performance as serological markers for the early diagnosis of gastric cancer. COL10A1 belongs to the collagen family, and its expression level in gastric cancer is closely related to the invasiveness and prognosis of the tumour [14]. By influencing the interaction between the extracellular matrix and immune cells, it promotes the invasion and metastasis of tumours [15]. Studies have shown that high expression of COL10A1 is associated with advanced-stage gastric cancer and may be related to the infiltration of immune cells in the tumour microenvironment [16][17]. Relevant studies [18] reported that the AUC of plasma COL10A1 in detecting gastric cancer patients was 0.9171, and the AUC value in detecting early gastric cancer was 0.8789. The performance was comparable to the diagnostic performance of serum COL10A1 in this study. GKN2 belongs to the gastric factor family and plays a vital role in maintaining the integrity and homeostasis of gastric cancer [19]. Studies on the role and mechanism of GKN2 in the proliferation and metastasis of gastric cancer cells have shown that GKN2 may be involved in the development of gastric cancer by affecting the proliferation and metastasis ability of gastric cancer cells [20]. Other studies have shown that the expression level of serum GKN2 in the gastric cancer group is greater than that in the normal control group and the precancerous lesion group [21].

Another study [22] reported that serum GKN2 and LIPF levels were regarded as predictors of gastric cancer in women. According to the performance study of the individual markers in this research, the performances of the three markers are all greater than those of the conventional markers PGI, PGII, and PGR. They show better performance in the detection of early gastric cancer than in the detection of gastric cancer and advanced gastric cancer [23][24][25].

This study revealed that the combined detection of COL10A1, LIPF and GKN2 can improve the diagnostic efficacy of individual markers. Correlation analysis showed a significant positive correlation among the three new markers. COL10A1 is related to the invasion and metastasis of gastric cancer cells, LIPF is related to metabolic changes in these cells, and GKN2 may be related to the proliferation and differentiation of gastric cancer cells. The combined detection of these factors can provide more comprehensive biological information on gastric cancer, thereby improving the accuracy of diagnosis [26][27][28]. However, the specific interaction mechanism among the three markers in the occurrence and development of gastric cancer has not yet been reported, and more basic research is needed in the future for in-depth exploration [29].

Furthermore, this study revealed that the combined detection of three new markers with traditional PGI and PGII can further improve the diagnostic performance for gastric cancer. The performance can be increased by 8.67% to 12.81% in the detection of gastric cancer patients at different stages. These findings suggest that 5MP can further supplement the detection performance of 3MP and PGR and significantly improve the diagnostic performance for gastric cancer.

In conclusion, COL10A1, GKN2 and LIPF are very promising serological markers for the early diagnosis of gastric cancer. The joint detection of the serum levels of these three markers, along with PGI and PGII, has significant reference value for the early diagnosis of gastric cancer. For high-risk patients, the combined detection of COL10A1, GKN2, LIPF, PGI and PGII, which can effectively improve the early detection rate and diagnostic efficiency of gastric cancer, is recommended.

## Dodatak

### Conflict of interest statement

All the authors declare that they have no conflict of interest in this work.
